# Perceptions of Social Mobility, Gender, and Progressive Politics

**DOI:** 10.1177/00104140241306939

**Published:** 2024-12-09

**Authors:** Briitta van Staalduinen, Delia Zollinger

**Affiliations:** 1 26567 University of Konstanz, Konstanz, Germany; 2 27217 University of Zurich, Zurich, Switzerland

**Keywords:** gender, social mobility, knowledge economy, political behavior, group politics

## Abstract

Extensive research explores the relationship between deepening conflict over socio-cultural issues and stagnating social mobility, typically focusing on men. Upwardly mobile women are routinely mentioned as belonging to the progressive “winners” of the knowledge-based society, yet their experiences and politics have received far less attention. This paper theorizes and investigates how women view their individual and collective trajectories and how these views relate to perceptions of future opportunities and political attitudes. Using survey data from four West European countries, we find that, while experiencing upward intergenerational mobility at the individual level is associated with positive opportunity perceptions, this relationship is not more pronounced among women than men nor linked to especially progressive attitudes. Rather, it is women sharing a sense of upward collective momentum who demand further action to achieve gender equality. This contrasts with men—including those perceiving themselves as upwardly mobile—who also acknowledge women’s collective gains: they more readily accept the still male-dominated status quo.

## Introduction

Recent decades have been marked by waning support for mainstream parties across Europe, which a growing body of research links to declining prospects for social mobility: those who experience stagnant or downward mobility are more susceptible to pessimism and nostalgia, less willing to adopt new social norms, and more likely to vote for far right parties (Burgoon et al., 2019; Engler & Weisstanner, 2021; Gest, 2016; Gest et al., 2017; Häusermann et al., 2023; Hochschild, 2016; Steenvoorden & Harteveld, 2018).

Yet, this prominent story primarily applies to the experiences of men. There are theoretical and empirical reasons for this. In theory, men—especially older, lower-educated men—are more likely than women to see their jobs and social position threatened by the shift towards a knowledge-based society. Empirically, the effects of downward mobility on support for radical right parties have been found to be weaker or absent for women (Bornschier & Kriesi, 2013; Gidron & Hall, 2017, 2020; Gingrich & Kuo, 2022; Kurer, 2020; Kurer & van Staalduinen, 2022; Mutz, 2018).

**Figure 1.**
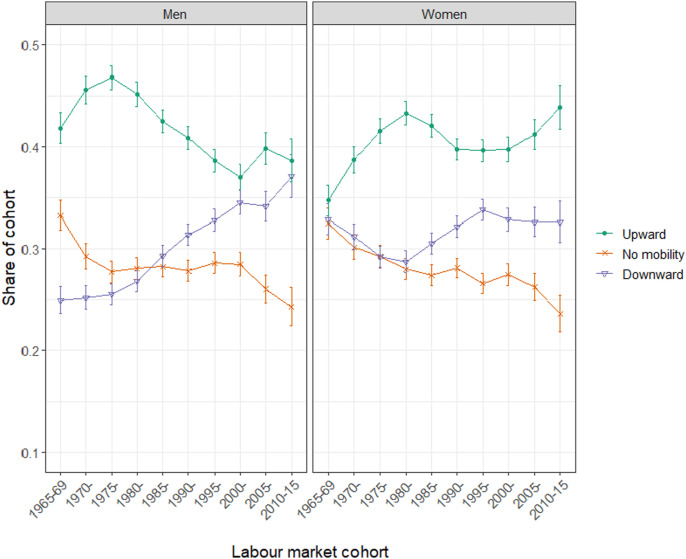
Class mobility by gender and labor market cohort. Note: The figure is adapted from van Staalduinen, (2022). It is based on a 15-country sample from rounds 1-9 of the European Social Survey (ESS). Mobility is calculated as the difference between the highest parental class and respondent class. Class is measured using the 5-category ESeC schema, which relies on ISCO codes and (necessarily) collapses differences between countries to facilitate cross-national comparisons. The *x*-axis refers to the year in which the group entered the labor market.

In fact, many scholars contrast the status losses of working-class men with the status gains experienced by women, emphasizing that, at least on some measures, women have been relative ‘winners’ of structural change. Empirical evidence points to substantial educational and occupational gains among women, including that they are more likely than men to acquire a university degree and less likely to experience long-term unemployment (Breyer et al., 2023; Garritzmann et al., 2022; Gidron & Hall, 2017; Iversen & Soskice, 2019). As shown in Figure 1, trends in social mobility among women improved significantly in the postwar decades and did so again more recently, buoyed by developments in the knowledge economy.

Given that women are also more likely to hold progressive socio-cultural attitudes and to support the ‘new’ left (Bornschier et al., 2021; Dassonneville, 2021; Hooghe & Marks, 2022; Iversen & Soskice, 2019), it is sometimes assumed that women’s status gains underpin gendered patterns in political attitudes and behavior. Much of the work examining ongoing political transformations of advanced democracies implies that upwardly mobile women are the political inverse, or ‘mirror image’, of downwardly mobile men, especially when it comes to socio-cultural conflicts: the disproportionate status losses experienced by men are linked to conservative backlash and women’s overwhelming status gains to progressive politics.

However, we know very little about the relationship between women’s status gains and their tendency to adopt progressive stances on socio-cultural issues. Do women adopt such stances in part because they as individuals have experienced the status gains facilitated by structural change? Or because they identify as members of a *group* favored by ongoing transformations in society? In elaborating on why women lean left, scholars tend to blur career trajectories facilitated by the knowledge economy with broader societal change fostered by socio-cultural movements and left-wing actors. As a result, existing work has yet to empirically explore the degree to which socially progressive stances among women are rooted in individual experiences of upward mobility or in a shared sense of collective momentum. This gap is especially surprising in light of extensive evidence that, in revealing the persistent barriers that even objectively successful women face in the labor market, cultural spheres, and politics (Goldin, 2021; Green & Shorrocks, 2023; Häusermann et al., 2015; Off, 2023), raises questions about how objective status trajectories manifest and matter subjectively.

In this paper, we start to address this gap by theorizing two distinct, though not mutually exclusive, accounts of women as progressive ‘winners’ in knowledge-based societies. The first version plays out at the *individual* level, with progressive attitudes among women reflecting the greater incidence of upward mobility in this group: women are confident about their opportunity prospects in society and socially progressive in their politics because they have experienced social mobility at higher rates than men. Whereas status anxieties have been linked to conservatism, women’s status gains instead make them at ease with and well-equipped to navigate social transformations, indeed having individually benefited from them. Importantly, gendered differences in progressive politics would be compositional here. Although women have experienced upward mobility at higher rates, from this theoretical perspective, it should have similar effects on men and women. In this account, then, the contrast between upwardly mobile women and downwardly mobile men is most important.

A *collective* account suggests a more directly gendered dimension to the politics of social mobility, wherein the status gains of women as a group are what matters. That is, women’s left-leaning politics on socio-cultural issues reflect decades-long efforts by progressive actors to guide structurally rooted societal transformations in favor of women and away from traditional power hierarchies that largely benefit men. Women who perceive this positive group-based momentum likely also support changes in favor of other disadvantaged groups given left-wing efforts to build a broad universalist agenda for social justice that links women’s status gains to those of minorities and LGBTQ + groups (Abou-Chadi & Hix, 2021; Frega, 2018; Kitschelt and Häusermann, 2024). In this account, the comparison to *upwardly* mobile men also becomes important. The divergent consequences of advances in gender equality may give rise to a form of male ‘apprehension’ (cf. Häusermann et al., 2023). That is, even men doing well may be less confident about their future status in light of changing gender relations and therefore less supportive of progressive policies than their female peers (Kosakowska-Berezecka et al., 2020).

In developing and testing these two accounts, we also consider the possibility that progressivism among women has little to do with social mobility or status—an outcome with its own political implications. As noted previously, there are various factors that may lead women to remain skeptical about their future opportunities or keep them from straightforwardly associating their career trajectories with progressive changes in society.

To explore these two accounts, we focus empirically on *perceptions* of social mobility, mirroring a subjective turn in recent work on (male) status threat (e.g. Engler & Weisstanner, 2021; Gest et al., 2017; Gidron & Hall, 2017; Hochschild, 2016; Kurer, 2020).^1^ Furthermore, although intragenerational mobility merits equal attention, we focus on intergenerational mobility, given that gender relations tend to change gradually. We draw on observational data from an online survey fielded in 2021 in the UK, Germany, Spain, and Sweden—countries with different welfare regimes and facing varying challenges regarding the achievement of gender equality. This survey, while not designed specifically with gender dynamics in mind, has the advantage of capturing individual *and* collective mobility perceptions, confidence about status prospects, and attitudes concerning gender and other socio-cultural issues that are of central focus in scholarship on knowledge society ‘losers’ and ‘winners’. We do not extend our analyses to party choice as it is these intervening attitudes on an increasingly politicized socio-cultural dimension of politics that are often assumed yet seldom examined.

Our findings suggest that, although women who experience *personal* upward mobility are optimistic about their status prospects, they are not more progressive than women who experience no mobility or downward mobility. Instead, women are more likely to be particularly progressive—and only on issues related to gender equality—when they have positive views of women’s *collective* trajectory. That is, women who view societal change as having favored women are more likely to demand even further action on gender equality. This relationship is especially noteworthy in comparison to upwardly mobile men who perceive the same advancements in favor of women. Whereas women who perceive these advancements demand *more* interventions to advance gender equality, this positive feedback loop seems to be absent, and even negative, among men who acknowledge women’s collective mobility. Overall, our findings highlight the importance of political mobilization around collective trajectories—not just for status-threatened men but also for women who stand to benefit from societal transformations.

## Women as Knowledge Society ‘Winners’

In the aftermath of Brexit, the first Trump presidency, and the rising success of numerous far-right parties across Europe, scholarly attention turned toward those ‘left behind’ by globalization and the transition to a knowledge-based economy. Consistent evidence linking a heightened risk of job loss and declining prospects for mobility to radical right politics has given rise to a consensus that status gains and losses are core to contemporary electoral cleavages (Anelli et al., 2021; Baccini & Weymouth, 2021; Gidron & Hall, 2017; Hochschild, 2016; Kurer, 2020; Kurer & van Staalduinen, 2022; Mutz, 2018).

The role that gender plays in this story is often taken for granted. It is widely recognized that women are less likely to vote for the far-right (e.g. Allen & Goodman, 2021; Betz, 2004; Bornschier & Kriesi, 2013; Gest et al., 2017). Yet, there has been limited inquiry into what this reveals about the relationship between mobility prospects and political behavior among women (but see Gingrich & Kuo, 2022). Similarly, although women are often discussed as knowledge economy winners (at least in relative terms) and as a driving force of progressive political change (cf. Iversen & Soskice, 2019), this research seldom examines how women themselves experience their status gains and whether these experiences partly underpin gendered differences in politics.^2^

Indeed, many structural and societal changes that have fundamentally transformed electoral landscapes in advanced democracies have disproportionately benefited women. Rapid educational expansion among women has outpaced that among men (Breen and Müller, 2020; Hooghe & Marks, 2022; Iversen & Soskice, 2019); investments in childcare and public services alongside growth in cognitively-demanding, interaction-oriented jobs have helped facilitate women’s entry into the workforce (Autor et al., 2003; Garritzmann et al., 2022; Kitschelt, 1994; Kurer & Palier, 2019; Oesch, 2006; Orloff, 1996); and increasing levels of education have strengthened progressive left-wing movements and, gradually, greater social liberalization, including on the topic of women’s rights and roles in society (Gidron & Hall, 2017). It is thus unsurprising that women are among the groups most widely perceived to have gained status in recent decades, both in the public (Breyer et al., 2023; Green & Shorrocks, 2023) and among scholars (Bornschier et al., 2021; Garritzmann et al., 2022; Gidron & Hall, 2017; Hall, 2022; Hochschild, 2016; Iversen & Soskice, 2019; Kitschelt & Rehm, 2022; Kriesi et al., 2008; Kurer & van Staalduinen, 2022).

However, the arguments in this literature tend to blur experiences of individual upward mobility with broader societal changes favoring women. Of course, some women may identify with both, but they are theoretically distinct pathways to progressive politics. If it is expanded educational possibilities, successful careers, and economic independence that encourage women to adopt progressive attitudes, then individual mobility matters for progressive politics (Dobbin, 2009; Hall, 2022; Iversen & Soskice, 2019; Kriesi et al., 2008). If it is instead their identification as members of a group that has advanced in society and that aspires for continued improvements, women’s support for progressive politics has a different proximate basis, rooted in socio-cultural movements that build on structural changes—a recent example being #MeToo—and efforts of political actors, such as those of the new left in the 1970s and 80s (Hooghe & Marks, 2022; Kitschelt, 1994; Kitschelt & Rehm, 2014; Oesch & Rennwald, 2018; Häusermann & Kriesi, 2015, also see Bergh, 2007).

Even where the discussion of women’s experiences is more extensive and careful (e.g. Gingrich & Kuo, 2022), we have yet to match the analytical clarity or empirical scrutiny that has accumulated on downwardly mobile men. Existing work not only blends individual and collective mechanisms, but also does not sufficiently account for the persistent structural barriers faced by women—including objectively successful women—and the ways in which these barriers may moderate any expected optimism about their status prospects. In the next section we offer a starting point for addressing these shortcomings by theoretically distinguishing between two conceivable mobility-based pathways to progressive politics among women. In doing so, we also discuss work offering various reasons why, for women, the link between social mobility and politics may be more tenuous.

## Women and the Politics of Opportunity

In an attempt to theoretically disentangle the blended array of claims about why women’s status gains underpin their affinity for progressive politics, we break down the attitudinal sequence outlined, sometimes only implied, in leading accounts of women as ‘winners’. We begin by reviewing explanations for why individual and collective mobility—understood broadly as the perceived trajectories of individuals or groups within social hierarchies—should relate to opportunity prospects (i.e. expressing confidence about the future as opposed to anxiety about status loss, cf. Mutz, 2018; Gidron & Hall, 2017; Gest et al., 2017; Häusermann et al., 2021). We then present explanations for why, due in part to the ways in which they instill greater optimism about the future, positive mobility perceptions should be linked to progressivism, both with respect to general socio-cultural issues and more specifically to issues relating to gender equality (i.e. the political opposite to traditionalist-conservative positions associated with status-anxiety, cf. Hochschild, 2016; Iversen & Soskice, 2019).

### Social Mobility and Opportunity Prospects

A first account of women as ‘winners’ operates at the individual level, contrasting the status anxiety of a male-dominated working class with the status optimism of highly educated female professionals. Due to structural transformations ongoing since the postwar era, the upwardly mobile in society are more likely to be women, while the downwardly mobile are more likely to be men. This compositional difference, in theory, suggests that women should be more optimistic about their future status prospects, though the *relationship* between social mobility and opportunity perceptions should be similar among men and women. That is, upwardly mobile men should be as optimistic as their female peers.

**H1a (compositional):** Perceptions of individual upward mobility are associated with confidence about future opportunities in the same way for women and men.

Although such a hypothesis may seem obvious in the context of research on knowledge economy ‘winners’, there are in fact numerous reasons for which labor market success may fail to instill confidence about future prospects among women. Recent research shows that women, regardless of education or occupation, are more vulnerable to new social risks, such as single parenthood or unequal ‘mental loads’ (Bonoli, 2005; Goldin, 2021; Weeks, 2024) and also more likely to be labor market ‘outsiders’ employed under precarious conditions and poorly protected by welfare states (Emmenegger et al., 2012; Häusermann et al., 2015). Furthermore, ‘child penalties’, inequalities in unpaid workloads, and other gendered wage gaps persist, including for women in secure emplyoment (Andreas Lagemann and Christina Boll, 2018; Cooke, 2011; Fortin et al., 2017; Hwang et al., 2023; Kleven et al., 2019; Togeby, 1994). The issues brought to the fore by the #MeToo movement (Archer & Kam, 2021) and the COVID-19 pandemic (Zamberlan et al., 2021) also shed light on why individual career trajectories may not be a straightforward source of status optimism among women.

This evidence in fact points to another line of reasoning whereby experiences of collective mobility may (also) matter for women’s opportunity perceptions. Regardless of the struggles or successes of individual careers, how women perceive their future prospects may depend on the degree to which they identify as members of a group benefiting more generally from societal change. That is, it may be less central whether women have objectively joined the ranks of ‘winners’, but rather whether the attention drawn to women’s collective gains has given them reason to remain hopeful about the future. Such a view is not only in line with accounts emphasizing how ongoing shifts in public discourse and mainstream media have ‘revealed the possibility of emancipation’ and heightened women’s aspirations (Hooghe & Marks, 2022, p. 5), but also with a growing emphasis on group-based perspectives in research exploring status loss (Anelli et al., 2021; Baccini & Weymouth, 2021; Bolet, 2021; Burgoon et al., 2019; Cramer, 2016; Engler & Weisstanner, 2021; Gidron & Hall, 2017; Hochschild, 2016; Kurer, 2020) and with recent work highlighting the importance of aspirational voters (Häusermann et al., 2021; Iversen & Soskice, 2019).

Of course, the extent to which women actually perceive such positive group momentum deserves scrutiny given the societal barriers discussed above and the sacrifices they require from women. However, one of the aims of cultural and political movements around gender equality—whether in addition to or as replacement for individual experiences—is to simultaneously recognize the gains made in the context of societal transformations and, in raising awareness about the gendered risks and barriers that women continue to face, emphasize that these gains are still insufficient (see, for instance Togeby, 1994, p. 217, or Hooghe & Marks, 2022, p. 5). In other words, women’s collective grievances often take the shape of recognizing change *and* demanding more of it.

In contrast to H1a, this collective account implies that women may not only differ from downwardly mobile men, but *also* from upwardly mobile men. If optimism about future status prospects is a gendered phenomenon and centered on group-based politics, even men who embrace women’s gains thus far may be apprehensive or have reservations about continued changes in gender hierarchies due to the implications these changes may carry for their own status.^3^

**H1b (gendered):** Perceptions of women’s collective upward mobility are more positively associated with confidence about future opportunities among women than among men.

### Social Mobility and Socio-Cultural Attitudes

Assumptions surrounding women’s optimism about their opportunity prospects are often extended to explain their support for socially progressive causes (e.g. Hooghe & Marks, 2022, pp. 5-6), though again the literature is not always explicit about whether the relevant processes are supposed to unfold at the individual or collective level. At the individual level, women’s progressivism could stem from them being direct beneficiaries of the transition to a knowledge-based society. In contrast to downwardly mobile men, whose experiences of status loss generate anxiety about the growing importance of education, diversity, and cosmopolitan values for career success, upwardly mobile women should not only feel at ease with but even enthusiastic about such changes (Iversen & Soskice, 2019; Kim & Hall, 2023). This embrace of change following their own upward trajectories should make women more likely than downwardly mobile men to adopt progressive attitudes, though again the *relationship* between mobility and these attitudes should then be the same among men and women. That is, in this account, men who experience upward career trajectories in the context of structural change would be just as likely to adopt progressive stances on socio-cultural issues.

**H2a (compositional):** Perceptions of individual upward mobility are associated with progressive socio-cultural attitudes in the same way for women as for men.

However, just as there were reasons to question individual-level accounts of women’s status optimism, women’s own mobility experiences may not be decisive for their progressive attitudes. Objective career success may obscure workplace experiences that, in generating ambiguity about one’s prospects, complicate the link between individual upward mobility and progressive politics. Women’s political attitudes may also be influenced by their partner’s status (Abou-Chadi & Kurer, 2021; Häusermann et al., 2016) or their attachment to conventional markers of social status may be weaker (Gingrich & Kuo, 2022).^4^ Furthermore, evidence suggests that upward mobility can give rise to a stronger belief in individual effort and merit (Lipset & Bendix, 1967; Mijs et al., 2022). Although much of this evidence is based on the experiences of men, women who see themselves as having succeeded *despite* persistent gendered societal barriers could be especially likely to adopt such beliefs (Lipset & Bendix, 1967; Mau, 2015), complicating their relationship to the progressive movements that often emphasize structural disadvantages.

These various strands of work draw our attention to how, beyond individual mobility experiences, how women view their trajectory as a *group* might also shape their orientation towards progressive politics. In fact, from a group-based perspective, theorizing the link between perceived mobility and progressive socio-cultural attitudes becomes significantly more straightforward and direct. The transition to a knowledge economy set in motion various societal transformations that were politically articulated and further advanced in successive waves of feminism, civil and gay rights activism, a growing environmentalist movement, and numerous global human rights initiatives that activated and strengthened ‘universalistic’ group identities, including those among women (Beramendi et al., 2015; Bornschier et al., 2024; Kriesi et al., 2008). As progressive left-wing actors have hailed and made visible improvements in the position of women in society, mobilizing them around a collective consciousness, they have also bundled women’s issues with the politicization of rights for minorities defined by race, immigrant status, or sexuality (Frega, 2018; Kitschelt and Häusermann, 2024).

It would then not necessarily be the direct individual economic and status gains offered by, say, expanding socio-cultural and cognitively-intensive professions but rather the ways in which women on knowledge economy trajectories are particularly likely to be mobilized around a sense of upward, if still constrained, collective momentum.^5^ If women are aspirational about the prospects of continued shifts in broader societal hierarchies, they will also be more likely than their male peers to demand further action to expand the rights of immigrants, LGBTQ+, and other disadvantaged minorities (cf. Bergh, 2007; Togeby, 1994, p. 234).^6^

As such, this collective account again raises the possibility that positive views of women’s collective mobility carry divergent consequences for socio-cultural attitudes among upwardly mobile men and women. This could apply to political demands more narrowly targeted at advancing women’s common interests, from childcare provision and gender quotas to equal pay legislation and tax incentives for dual-earner families (Folke & Rickne, 2020) or more broadly to interventions designed to challenge traditional social hierarchies. In contrast to women who, recognizing collective gains, are more likely to endorse demands for further improvements in favor of women and other disadvantaged groups, men—including upwardly mobile men—who acknowledge women’s gains should be relatively less likely to embrace continued changes to a social order from which they tend to benefit, whether that manifests in more moderate support for social change or in outright hostility to it.

**H2b (gendered)**: Perceptions of women’s collective upward mobility are more positively associated with socially progressive attitudes among women than among men.

Note again that, while our main goal is to disentangle and study these two quite distinct accounts of women as progressive ‘winners’ in knowledge-based societies—namely the compositional (individual) account versus the gendered (collective) account—we do not view them as necessarily mutually exclusive, and in section 5 we consider how they might interact. At the same time, in light of the continued precarity and discrimination that many women experience, we want to emphasize that, given its prominence in the literature examining status loss among men (which is often *not* rooted in objective individual material decline), it is important to consider the same group-based perspective in relation to women’s experiences, in both political science scholarship and the public debate more broadly.

## Data and Measurement

To examine these hypotheses, we draw on data from an online survey fielded in spring 2021 in Germany (*n* = 3000), Sweden (*n* = 3000), Spain (*n* = 1500), and the UK (*n* = 1500).^7^ The survey included population-representative quotas for age, gender, education, and labor market status, and the social research company Bilendi implemented the fieldwork. While the questionnaire was not designed primarily with social mobility and gender dynamics in mind but rather with social policy preferences and political attitudes more broadly, various items allow us to explore the relationships of interest. Most importantly, the survey includes individual and collective measures of mobility, an item on respondents’ perceived prospects in a fast-changing society, and a range of political attitudes. We introduce these measures while also discussing their limitations.

### Dependent Variables

For the first dependent variable, opportunity perceptions, we use an item asking respondents to evaluate their future employment prospects given ongoing societal changes. More specifically, respondents are asked, *“The world is changing fast. If you think of your future, how do you rate your personal chances of being in good, stable employment until you will retire?”*, with responses ranging from zero (very bad) to 10 (very good). This item can be seen as capturing the extent to which respondents see a secure position for themselves in a transforming society. A long-term labor market perspective measured in terms of stability may be considered particularly relevant from a gender perspective.

For the attitudinal outcomes, we first consider social progressivism in a broad sense. To capture positions on socio-cultural issues, we use three available survey items gauging respondent support for immigration, EU integration, and gay adoption (i.e., items routinely associated with the socio-cultural, or ‘second dimension’, of politics, Häusermann & Kriesi, 2015; Oesch & Rennwald, 2018). Respondents are presented with a statement on each issue (e.g. “Immigration is a threat to the national labour market”) and then asked to indicate their agreement with the statement, from 1 (disagree strongly) to 4 (agree strongly).

Second, to measure progressivism specifically on issues relating to gender, we use three available survey items that ask respondents about their stance towards feminism and about their support for expanding public childcare. The first of these items asks respondents how close they feel to feminists on a 1 (not at all close) to 10 (very close) scale, allowing respondents to express identification with but also distance themselves from this label. A second item asks whether the government should expand or reduce childcare services (1 = reduce, 7 = expand). The third item captures the degree to which the expansion of childcare is a priority, asking respondents “how urgent” they consider expanding “access to good-quality childcare services” (1 = not at all urgent, 4 = very urgent). This last item aims to identify potential differences among respondents who otherwise endorse the expansion of public childcare.

Regarding the items relating to gender, a tailored survey would have ideally included a more targeted range of items, for instance, on modern sexism (Anduiza & Rico, 2022; Swim et al., 1995), gender discrimination (Green & Shorrocks, 2023), additional policies relevant to gender equality (e.g. quotas; Einarsdóttir et al., 2020; Weeks, 2022), or on more intersectional perspectives (cf. Off, 2024). Looking at the data available, we consider feminist identity and support for/prioritization of childcare an especially hard test for finding differences between men and women; in the first case because even many progressive women might hesitate to call themselves feminist;^8^ and in the second because men also stand to benefit from high-quality childcare services. Put differently, if we find gendered patterns in how perceptions of mobility relate to support for childcare, we could also expect to find them if we looked at quotas or other policy measures seen as heightening competition from women for jobs, promotions, etc.

### Independent Variables

**Figure 2.**
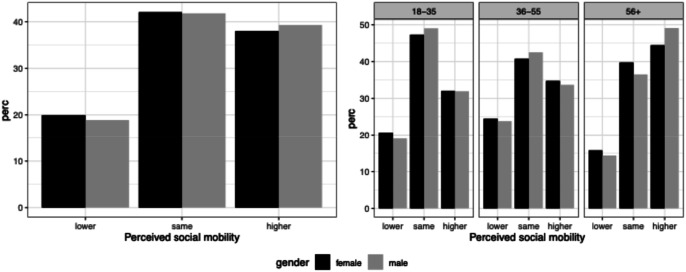
Distribution of individual mobility perceptions compared to parents.

For the first independent variable of interest, individual social mobility, we rely on perceived social mobility relative to one’s parents. Respondents are asked, *“If you compare your position in society with the one your parents had at your age. Would you say that your position now is lower or higher compared to their position back then?*” The same question is also asked with respect to respondents’ mothers.^9^ Figure 2 and Figure A.1 show the distribution of thes variables by gender (left panel) and by gender and age (right panel). In terms of overall patterns, Figure 2 shows that, while the majority of respondents perceive themselves as occupying either the same or higher positions than their parents, the share of those perceiving themselves as occupying the same position has been increasing across cohorts. As we might expect, fewer younger respondents see themselves as upwardly mobile. Appendix Figure A.1 shows that mobility perceptions relative to mothers are more positive. This can be taken as evidence that women’s status in the past is perceived to be lower than men’s and lower than that of subsequent generations of both men and women.

When it comes to gender-specific patterns, there are overall limited differences between men and women, including regarding perceptions of social mobility relative to mothers. That is, when prompted to consider the position of their mothers, women do not view their mobility trajectories differently from men. Overall, the higher rates of objective upward mobility among women that have been documented elsewhere do not seem to be fully reflected in how women themselves perceive their status trajectories. This casts initial doubt on the compositional account of women perceiving themselves, individually, as winners. Figure B.1 in the Appendix, showing perceived social mobility by country, points to broadly the same pattern between men and women across the four countries. Although women in Spain perceive themselves as overall less mobile than women elsewhere, so do Spanish men relative to men elsewhere. These cross-national patterns in perceptions of individual mobility, then, indicate that while our measure is subjective, it also reflects the ways in which structural transformations condition people’s perceptions; we would expect Spain to have overall lower levels of upward mobility given occupational trends among younger cohorts in recent decades (De Pablos Escobar & Gil Izquierdo, 2016).

**Figure 3.**
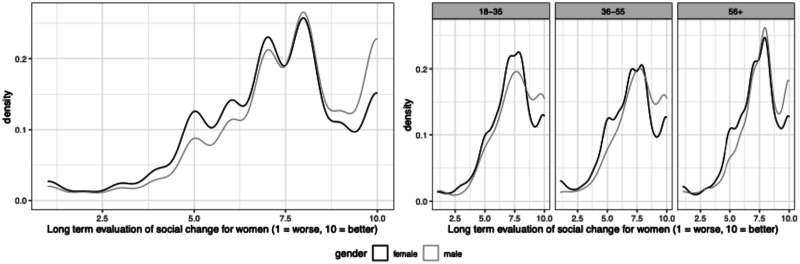
Distribution of perceived collective gains for women.

For the second independent variable of interest, the collective mobility of women, we use an item capturing perceived societal gains for women as a group. Respondents are asked, *“Would you say that life for women in [COUNTRY] has become better or worse than 30 years ago?”*, with answers ranging from 1 (worse) to 10 (better). The survey also includes a question about whether life in the survey country has in general become better or worse, which we later use as a benchmark.^10^ Figure 3 shows the distribution of responses to the question about women’s trajectory by gender and age. Overall, life for women is perceived to have improved substantially over recent decades. Importantly, men tend to see greater improvements in women’s lives than women do themselves, and this pattern is strikingly consistent across age groups and countries (see B.2 in the Appendix). A more tailored survey ideally would have asked specifically about various aspects of women’s lives or even solicited an explanation of respondents’ assessments. Notwithstanding these limitations, the item allows for an initial comparison of how individual and collective group trajectories relate to a series of political variables.

### Socio-Demographic and Structural Correlates of Mobility Perceptions

Given the characteristics of deindustrialization and the transition to a knowledge-based economy, there are certain socio-demographic factors that we would expect make some women more likely to perceive themselves as having gained than others. To explore heterogeneity among women, section C in the Appendix provides information on the socio-demographic predictors of individual mobility perceptions as well as perceptions of women having made collective gains. In general, the subgroups of women understood as relative knowledge society winners in prevailing scholarship are indeed those who perceive themselves and/or their group as upwardly mobile. More specifically, highly-educated women working as managers, sociocultural professionals and technical professionals in full-time employment perceive both their own trajectory and that of women more favorably. In stark contrast, women with lower levels of education employed part-time in the service industry and in production work evaluate both their own and women’s trajectory more negatively. This heterogeneity highlights that, while women have made significant gains in fast-growing sectors, they also continue to struggle in more precarious areas of the labor market—a pattern that aligns with work emphasizing the barriers that women continue to face (Emmenegger et al., 2012; Häusermann et al., 2015). Apart from these main factors, favorable perceptions are more pronounced among women who report similar earnings as their partners—a pattern notably reversed among men. Having children, meanwhile, is positively associated with perceptions of status mobility among men but not women.

As we move on to analyzing how mobility perceptions relate to attitudes, these descriptive analyses give an idea of which kinds of individuals have favorable views of their own mobility and of women collectively. They also help to validate our approach of capturing perceptions of individual and collective mobility. Our measures are subjective but clearly not divorced from the structural and societal transformations discussed above. In subsequent analyses we intentionally do not control for all of these factors. Based on our theoretical framework, it does not make sense to try to isolate a clean, net effect of mobility perceptions. We think of such perceptions as strongly determined by socio-structural circumstances, material realities, and socializing environments. We do, however, control for age and education, given well-established evidence that they are central to experiences of social mobility and to sociocultural attitudes. While this means that we include a variable that is related to class—see e.g. Iversen and Soskice (2019); Abou-Chadi and Hix (2021); Beramendi et al. (2015); Marks et al. (2022) on the direct importance of education for life chances and politics in the knowledge society—opportunities still vary significantly within education groups, including among younger cohorts (Ansell & Gingrich, 2017). Conceptually, then, our interest will be in the gendered associations between mobility perceptions and attitudes, holding age and education constant.

Before we turn to discussing the results, it is worth reiterating that our data is observational. This is well-suited to the aims of this paper: our goal is to theoretically spell out and start to empirically disentangle two plausible relationships between social mobility perceptions and progressive politics that are often implicit and/or blurred in existing scholarship. Our theoretical framework does not imply a neat, unidirectional causal effect of ascent in the knowledge economy, but rather recognizes the importance of progressive mobilization efforts, especially for the gendered, group-based mechanisms we propose. Nor does it ignore the possibility that these two sources of women’s left-leaning politics may interact and reinforce one another over time. Our aim, rather, is to theoretically and observationally distinguish two conceivable links between mobility perceptions and progressive politics—only one of which leads us to expect an interactive relationship with gender.

## Results

### Social Mobility and Opportunity Perceptions

We test our hypotheses using OLS regressions, each model including country fixed effects as well as controls for age and education. The coeffcient estimates we are particularly interested in are those for the interaction of gender with the two independent variables listed above: perceptions of individual and collective trajectories. Table 1 provides an overview of our hypotheses, the variables used to operationalize key concepts, and our main findings.

**Table 1.** Summary of Hypotheses, Variables & Findings.

Hypothesis	Independent variable	Dependent variable	Finding
H1a: Individual mobility & opportunity perceptions: Positive relationship, not gendered	Societal position relative to parents	Personal chances of being in good, stable employment until retirement	Positive relationship, not gendered
H1b: Collective mobility & opportunity perceptions: Positive relationship, gendered	Life for women better or worse than 30 yrs ago	Personal chances of being in good, stable employment until retirement	Positive relationship, gendered
H2a: Individual mobility & social progressivism: Positive relationship, not gendered	Societal position relative to parents	**General:** Support for immigration, EU integration, adoption rights **Gender:** Support for feminism, childcare	**General:** No relationship
			**Gender:** No relationship
H2b: Collective mobility & social progressivism: Positive relationship, gendered	Life for women better or worse than 30 yrs ago	**General:** Support for immigration, EU integration, adoption rights **Gender:** Support for feminism, childcare	**General:** No relationship
			**Gender:** Positive relationship, gendered

**Figure 4.**
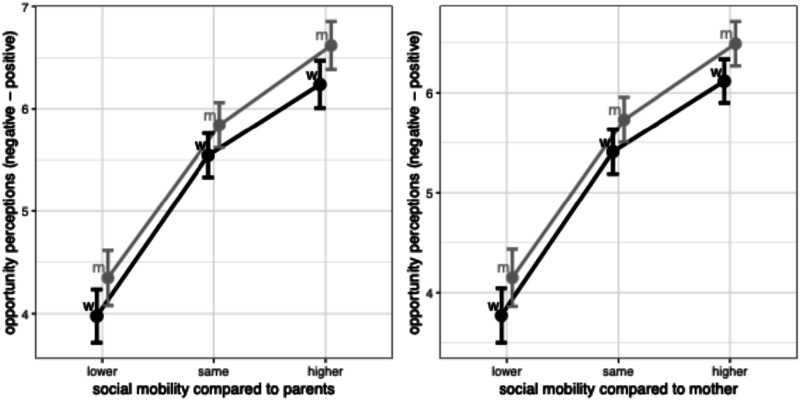
Perceptions of individual upward mobility positively associated with opportunity perceptions. Note: OLS regressions controlling for age and education, with country FE.

The first set of results concerning opportunity perceptions support hypotheses H1a and H1b. Figure 4 shows that women who perceive individual upward mobility are as likely as men who perceive individual upward mobility to be confident about their future opportunities.

This suggests that confidence about their prospects vis-a-vis downwardly mobile men are at least in part compositional. We can read this as evidence that women might extrapolate from experiences of individual mobility to beliefs about future opportunities in ways similar to men, although, if anything, the results point towards a stronger positive association for men. Even when considering a comparison to mothers (right panel in Figure 4), which could be expected to matter more for women, there is no gendered relationship.

**Figure 5.**
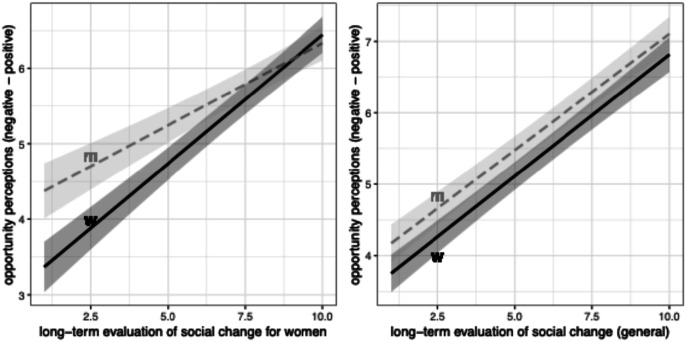
Views of women’s collective upward mobility positively associated with opportunity perceptions *especially* among women (more so than among men). Note: OLS regressions controlling for age and education, with country FE.

At the same time, women who view societal changes as favoring women are also more likely be confident about their status prospects, and, consistent with H1b, this relationship is stronger than for men (left panel in Figure 5; the estimate for the interactive coeffcient is significant at the .001 level). As shown by a comparison between the left and right panels in this figure, this interactive result is specific to evaluations about societal developments for women; there is no interaction when we consider evaluations of social change in general. We can interpret this as support for the idea that collective trajectories matter for gendered differences in confidence about opportunity prospects: the flatter upward slope for men suggests that, although men who recognize improvements in women’s lives also tend to be more confident about their own prospects (and, we might interpret, more at ease with societal change in general), this relationship is even more pronounced among women. In other words, beyond reinforcing existing evidence that individuals who accept the direction of societal transformations are generally less anxious about their own future, these results show that individuals who view their group as benefiting specifically from these transformations—in this case women—are especially confident about the future.

Table D1 in the Appendix shows the full regressions underlying Figures 4 and 5. Two additional models further show that the interaction effect between views of changes in women’s lives and gender holds when we control for individual mobility experiences, whether compared to parents or to mothers. Net of the positive effects of individual upward mobility trajectories, perceiving a collective upward trajectory for women is positively associated with confidence about future opportunities, and more so among women than among men.

### Social Mobility and Socio-Cultural Attitudes

**Figure 6.**
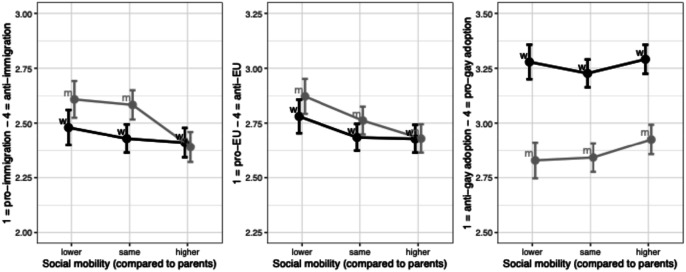
Perceptions of individual upward mobility not associated with general progressivism among women. Note: OLS regressions controlling for age and education, with country FE.

When it comes to the second set of hypotheses, we find support for our expectation about a gendered link between women’s collective gains and progressive attitudes (H2b) but only when it comes to issues relating specifically to gender equality. Contra to our hypotheses, status gains at the individual level are not clearly associated with general progressivism among women (H2a), nor is *general* progressivism particularly pronounced (relative to men) among women who perceive collective gains (Figure 6 and Figure D.2, full regression results shown in Appendix Tables D2 and Tables D5). These results challenge the idea that women’s affinity for progressive causes can be attributed to individual trajectories in the knowledge economy, as well as the assumption that their progressivism reflects a sense of shared purpose with other disadvantaged segments of society—a purpose that would be expected to set them apart from men. Instead, we might read these results as suggesting that socio-cultural movements and political efforts drawing *specific* attention to women’s collective trajectory and related gender issues seem to play an important role.

To examine these results in more detail, we begin by highlighting the absence of clear relationships between individual mobility and broad socio-cultural progressivism among women, including in comparison to men (Figure 6; recall that we did *not* expect an interaction with gender at this individual level). In line with existing work (Kurer & van Staalduinen, 2022), we find some evidence of socio-cultural conservatism among downwardly mobile men who are more intensely anti-immigrant than upwardly mobile men; suggestive but not significant evidence for attitudes towards the EU points in a similar direction. Meanwhile, across the three issues, there is no link between individual mobility and progressive stances among women, indicated by the relatively flat slopes in all three panels of Figure 6. Upwardly mobile women also appear no more feminist than women on downward or stable trajectories (Figure D.1). Taken together, these results run counter to H2a and suggest that perceptions of individual mobility trajectories have little bearing on progressive attitudes among women.

As discussed in Section 3, there are various reasons why individual upward mobility may matter little for progressivism among women. Women may be politically less sensitive to their individual status trajectories, the status markers inherent in comparisons to parents (occupation? Income? Breadwinner role? Home ownership?) being less relevant or salient to women than they are to men. Women’s objective ascent in the knowledge economy may also obscure workplace experiences and career trajectories that, for a wide range of reasons, complicate their relationship to progressive politics. Although, as mentioned previously, existing research raises meritocratic beliefs as an obvious mediating factor, Figures E.1 and E.2 in Appendix E show that the results for women (and men) hold when the models include an item indicating belief in the importance of hard work for “someone getting a well-paid job”. This suggests that the relationship between individual upward mobility and political attitudes is not as uniform as either meritocratic frameworks or ‘winner’ accounts suggest.

**Figure 7.**
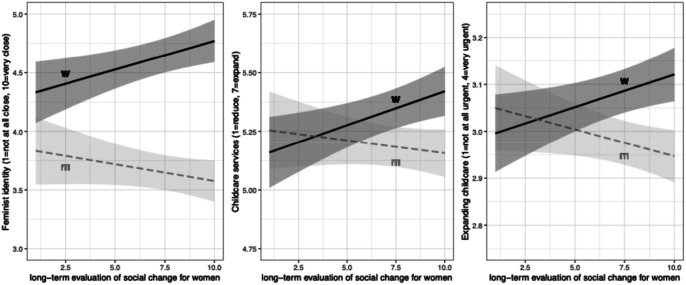
Views of women’s collective mobility positively associated with progressivism on gender equality among women (but not among men). Note: OLS regressions controlling for age and education, with country FE.

While our results do not suggest that individual upward mobility contributes to explaining differences in socio-cultural attitudes, we do find limited evidence consistent with H2b. Positive views of women’s upward collective trajectory are associated with more unity around issues related to gender equality (Figure 7; full regression results in Appendix Tables D4 and D3). Furthermore, consistent with our interactive hypothesis H2b, although the effects are

Small, the estimates for each of the three interactive coeffcients are significant at the .01 level and indicate that perceiving society to be changing in favor of women relates differently to identification with the feminist movement and to gender-related policy preferences among women than it does among men. Not only is the upward slope for women steeper, with female respondents perceiving gains for women also likely to demand further action, but the slope for men in Figure 7 trends downwards across all three panels: men who perceive positive societal changes in favor of women seem, if anything, less likely to embrace further action to promote gender equality.

Table D6 in the Appendix shows that the interaction between perceptions of women’s collective trajectory and gender is robust to the inclusion of perceptions of individual social mobility, as was also the case for the interaction effect on opportunity perceptions observed in Figure 5 (see Table D1). The interactive effects on closeness to feminists and prioritization of expanding childcare (though not on general endorsement of childcare expansion) also hold when the models are re-estimated using a sample that excludes the downwardly mobile (not shown). In short, among the upwardly mobile, men and women appear to diverge in their views on whether society has sufficiently progressed when it comes to gender equality.

These patterns serve to illuminate nuances in progressivism among women and men, suggesting that they are far from a consistent bundle of attitudes. That women who perceive women’s collective gains positively are not *particularly* progressive (relative to men) on other socio-cultural issues challenges portrayals of women’s automatic support for causes that champion disadvantaged groups (even when women are mobilized “feminists”). Furthermore, men who perceive women’s lives as improving and who are as likely as women to adopt progressive positions on immigration or EU integration (Figure D2) do not adopt similarly progressive positions on issues relating to gender equality (Figure 7). These results carry numerous implications. First, they call for more attention to variation in positions across issues typically grouped as progressive, especially where gendered mobility trajectories and socio-cultural conflicts are concerned; progressive positions on one issue do not implicate corresponding positions on others. Furthermore, these results suggest that, whereas positive views of collective mobility among women may generate a positive feedback loop whereby they demand further change, such views among men seem instead associated with weaker demand for equalizing interventions and greater acceptance of the status quo.

As noted previously, the individual and collective accounts we have proposed are not necessarily mutually exclusive and there may even be reason to expect perceptions of individual and collective mobility trajectories to have an interactive effect on opportunity perceptions and political attitudes. The nature of the interaction, though, is not self-evident. On the one hand, it could be a reinforcing dynamic whereby positive views at one level reinforce positive views at another. On the other hand, we could also imagine a cross-cutting effect, with experiences at the individual level eroding, or perhaps complicating, the effects of perceptions at the group level. Using a *women-only* sample, we re-estimated the models with an interaction term for individual and collective mobility. The results, shown in Table D7, do not indicate a strong or consistent interactive pattern. Looking at feminist identification, there is weak evidence of a cross-cutting relationship: women who perceive improvements in women’s lives while *also* perceiving themselves to be upwardly mobile are rather less likely to identify as feminist. Given the results shown in Figure E.2, a fruitful area of inquiry for future research would be to explore how, if not for a stronger commitment to meritocracy, upward trajectories in the knowledge economy shape women’s orientation to feminism.

On the whole, our evidence can be read as pointing to the importance of group-based perceptions and to the ways in which political actors mobilize groups around the consequences of structural transformations, shaping how these transformations are perceived and translated into politics. In this sense, our findings also speak to older work highlighting the importance of feminist consciousness for understanding gender gaps in politics, always against a backdrop of structural change (e.g. Bergh, 2007; Cook & Wilcox, 1991; Manza & Brooks, 1998; Togeby, 1994). Our results indicate that, among women, perceiving individual upward mobility does not per se relate to progressive social positions. Perceiving gains for women as a group is more likely to do so. But even here, while men’s status anxieties have been shown to relate to broader social conservatism, a shared sense of status momentum among women is not automatically linked to broader social progressivism.

## Conclusion – Women as the ‘Mirror Image’?

The literature on electoral changes in the knowledge economy tends to associate women’s status gains in recent decades with conflicts over socio-cultural issues. However, the attention has been disproportionately directed at backlash among men, leaving the role of women’s own status perceptions in these conflicts largely unexplored. We know that the shift towards a knowledge-based economy has benefited only some subgroups of women and only on certain dimensions, raising questions about the extent to which women perceive themselves as winners. More generally, we lack a thorough theoretical and empirical understanding of how women’s supposed status gains relate to their political attitudes. We drew from existing scholarship to glean two accounts of how upwardly mobile women might in theory be the progressive ‘mirror image’ of downwardly mobile men. Focusing on the opportunity perceptions and socio-cultural attitudes that are widely viewed, if not always empirically tested, as intervening factors in the ‘funnel’ linking shifts in status hierarchies to voting behavior, we then explored these two accounts using observational survey data from four Western European countries.

A first account emphasizes that women have experienced paths to upward mobility in the knowledge economy at higher rates than men. On average, and *not* by virtue of their gender, they should thus be more confident about their future status prospects and more socially progressive. We find some support for this compositional story, in the sense that perceiving individual upward mobility is related to confidence about future opportunities in the same way for men and women. However, experiences of individual social mobility, at least measured in comparison to parents, appear unrelated to socially progressive attitudes among women.

A second account suggests a more directly gendered story about women who identify as members of a group experiencing a collective upward trajectory, which generates confidence in their future prospects as well as support for progressive causes. We find that a collective perspective does relate to women’s opportunity perceptions and that women who perceive their group to be gaining ground are more likely than men—including upwardly mobile men—to be mobilized around further demands for gender equality, as our theory led us to expect. We find no such interaction regarding other progressive issues.

Taken together, these results cast doubt on a supposed link between upward mobility, whether individual or collective, and general progressivism among women. While existing scholarship associates men’s relative status losses with a broad conservative backlash, here, the “reverse” link between women’s status gains and progressive politics appears to be more tenuous. There could be various reasons for this including that, as our analyses indicate, women who see themselves as upwardly mobile come from heterogeneous backgrounds and have charted different kinds of careers—as managers, in tech, or in public sector jobs (as we note in FN5, from a *mobility* perspective, expectations regarding such occupational profiles are far from clear-cut). Furthermore, beyond the challenge of rallying women to view the world through a gendered or group lens, mobilizing them to *also* endorse a more comprehensive universalist agenda spanning minority rights may be less automatic than is sometimes assumed or implied.

Our findings do suggest that political unity among women seems most likely to emerge among those who share a sense of collective status gains and only quite specifically for issues relating to gender equality. This lends evidence to an account of women’s progressivism that is conditional on the efforts of political actors to draw attention to and normalize discussion around the challenges that women face in a transforming society and to mobilize women to see politics as the arena in which to address these challenges. Complementary to this main finding is evidence of unease surrounding continued efforts to advance gender equality among (even upwardly mobile) men. Men—at least on our measures—are more likely to indicate that social changes in favor of women have advanced far enough, regardless of individual trajectories (see also Green & Shorrocks, 2023, who find evidence of gender backlash across income and educational levels).

We view our observational analyses as a starting point for exploring women’s perspectives in the politics of social mobility, especially given the wide array of factors that condition the relationship between educational and occupational trajectories, gender, and voting (Estevez-Abe, 2005; Inglehart & Norris, 2003; Iversen & Rosenbluth, 2010). Future research should aim to better understand how women define and benchmark their own status or mobility as compared to men, how various social environments (educational, occupational, etc.) influence women’s interpretations of their own and other women’s trajectories, and how the national party landscape and policy context play into women’s assessments of their trajectories in a transforming society—both individually and collectively.

## Supplemental Material

Supplemental Material - Perceptions of Social Mobility, Gender, and Progressive Politics Supplemental Material for Perceptions of Social Mobility, Gender, and Progressive Politics by Briitta van Staalduinen, and Delia Zollinger in Comparative Political Studies

## Data Availability

Replication materials and code can be found at van Staalduinen and Zollinger (2024).

## Notes

1. This shift arose largely due to the evident limitations of explaining far right support by looking narrowly at people’s immediate material well-being (cf. Häusermann et al., 2023, pp. 2-3). However, the resulting body of literature does not lose sight of how objective structural conditions (e.g. deindustrialization) create openings for political mobilization around status and identity-based grievances (cf. Anelli et al., 2021; Baccini & Weymouth, 2021; Kurer, 2020). While existing work pays particular attention to how such pressures might be experienced through a male breadwinner and/or racial lens, we take these same structural transformations as a backdrop for theorizing and examining women’s experiences (also see earlier work by e.g., Togeby, 1994).

2. While the literature on gender and vote choice pays much more attention to women’s experiences than research on knowledge economy winners and losers, the former stream of scholarship often focuses on how women’s objectively precarious labor market positions and immediate material circumstances shape their attitudes towards the left, especially through preferences for redistribution (Iversen & Rosenbluth, 2010; Shorrocks, 2018; Shorrocks & Grasso, 2020). We draw on this literature below. However, our interest is in extending the aforementioned ‘subjective turn’ in research concerned with deepening socio-cultural conflicts over identity and status to explicitly focus on women.

3. Research in social psychology suggests that men are especially likely to adopt zero-sum views of societal hierarchies and that these views intensify when they perceive increasing bias against them (Wilkins et al., 2015). Recent evidence indicates that such perceptions are increasingly widespread (Anduiza & Rico, 2022; Green & Shorrocks, 2023; Kosakowska-Berezecka et al., 2020).

4. We know that, in the past, women’s roles as mothers and caregivers had pronounced influence on their political attitudes, crowding out effects of labor market status (Bolzendahl & Myers, 2004; Shorrocks, 2018).

5. Be it experiences in female-dominated professions in the social and education sectors *or* exposure to male-dominated environments and glass ceilings in private tech or business sectors, women charting careers in the knowledge economy could be seen as especially receptive to collective new left mobilization. The point we want to stress is that mechanisms rooted in collective mobility should play out differently for women than for men, including women and men in similar occupations.

6. For a related argument about how far right voters connect arguments about group (dis)advantages based on gender, immigration, or LGBTQ + rights, see Off (2024).

7. The survey was developed in the context of the WELFAREPRIORITIES project, funded by the European Research Council (ERC) under the European Union’s Horizon 2020 research and innovation programme (grant agreement No 716,075). We thank Silja Häusermann, the PI of this ERC project, for generously providing access to the data.

8. This is evidenced by the low baseline levels of closeness to “feminists” in our survey (mean of 5.1 on a 1-to-10 scale among women; 4.1 among men). This may also reflect diffculties that political actors face in their efforts to build a unified feminist movement capable of speaking to women with very diverse experiences.

9. While the comparison to mothers suits our research interest and expands on existing studies that rely on fathers as reference points, unfortunately, the survey provides no information on the criteria by which respondents’ assess intergenerational mobility, whether relative to fathers or mothers (income? Occupational status?) As we discuss in Section 5, this could in principle vary by gender.

10. There is a concern that the measure for collective mobility at our disposal could be interpreted by respondents as asking them to *normatively* evaluate whether changes in women’s lives are positive or negative, meaning that it could covary with some of our outcome variables as a measure of progressivism. While future survey work on women’s perceived trajectories can and should improve on this (see also FN8), we take various steps to address these concerns: we use evaluations of *general* social change as a benchmark in our analyses, we show descriptively that women perceive less progress for women than men do (whereas a normative measure would be expected to yield more positive evaluations among women), and, in Section 5.2, we discuss why our findings validate this measure as capturing perceptions of women’s group trajectory rather than (just) progressivism. More specifically, we find an interactive relationship between this measure and gender, whereas a pure measure of progressivism should not behave differently for men and women.
