# Supplementary material for: Perceptions of Social Mobility, Gender, and Progressive Politics
Source: Comp Polit Stud. 2024 Dec 9;58(12):2718–49. doi: 10.1177/00104140241306939 (PMC12398354; doi:10.1177/00104140241306939)
Supplement: Supplemental Material - Perceptions of Social Mobility, Gender, and Progressive Politics [file sj-pdf-1-cps-10.1177_00104140241306939.pdf]

Appendix

A. Individual mobility perceptions compared to mothers

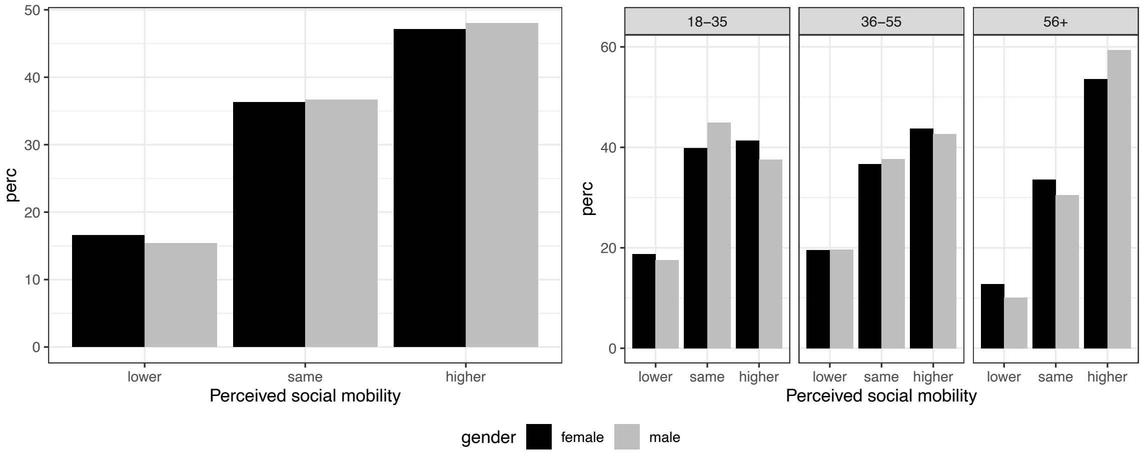

Figure A.1: Distribution of individual mobility perceptions compared to mothers

B. Social mobility perceptions by country

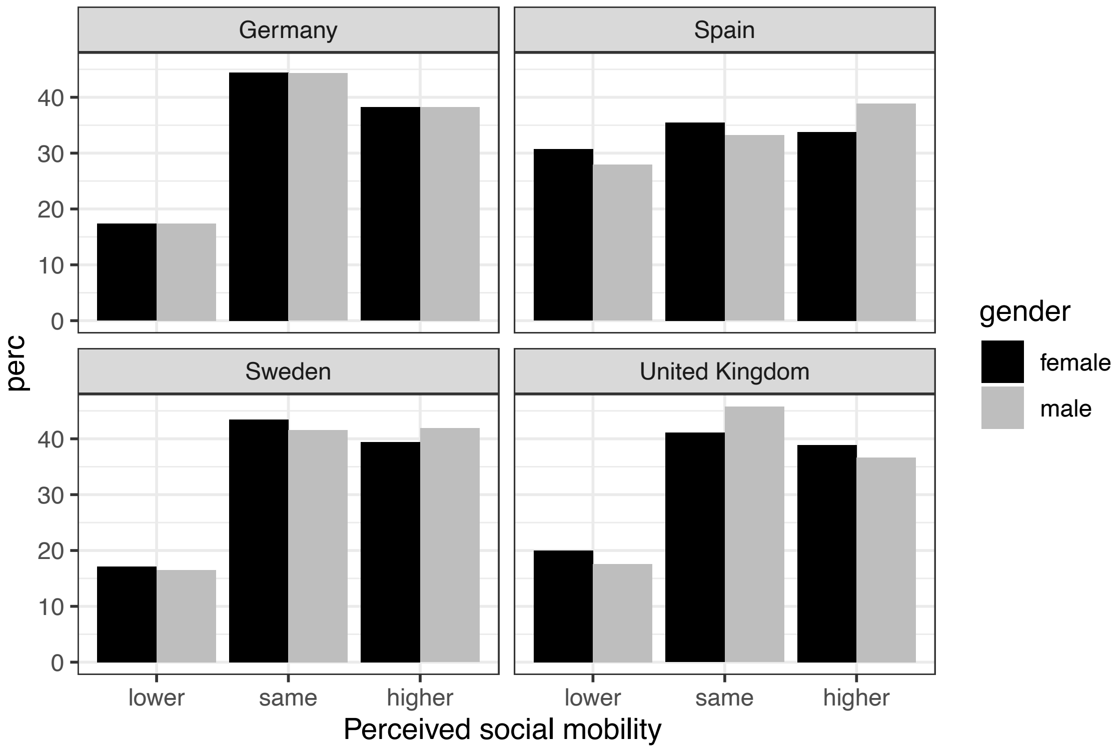

Figure B.1: Social mobility by country

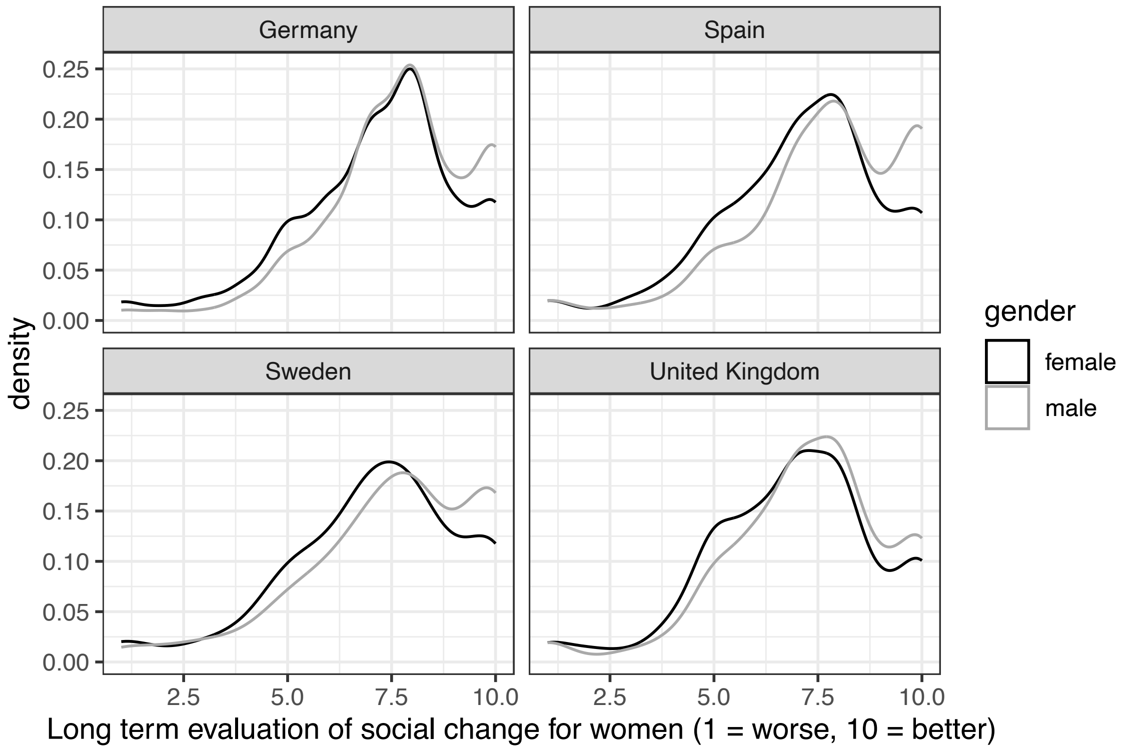

Figure B.2: Evaluations of change in favor of women

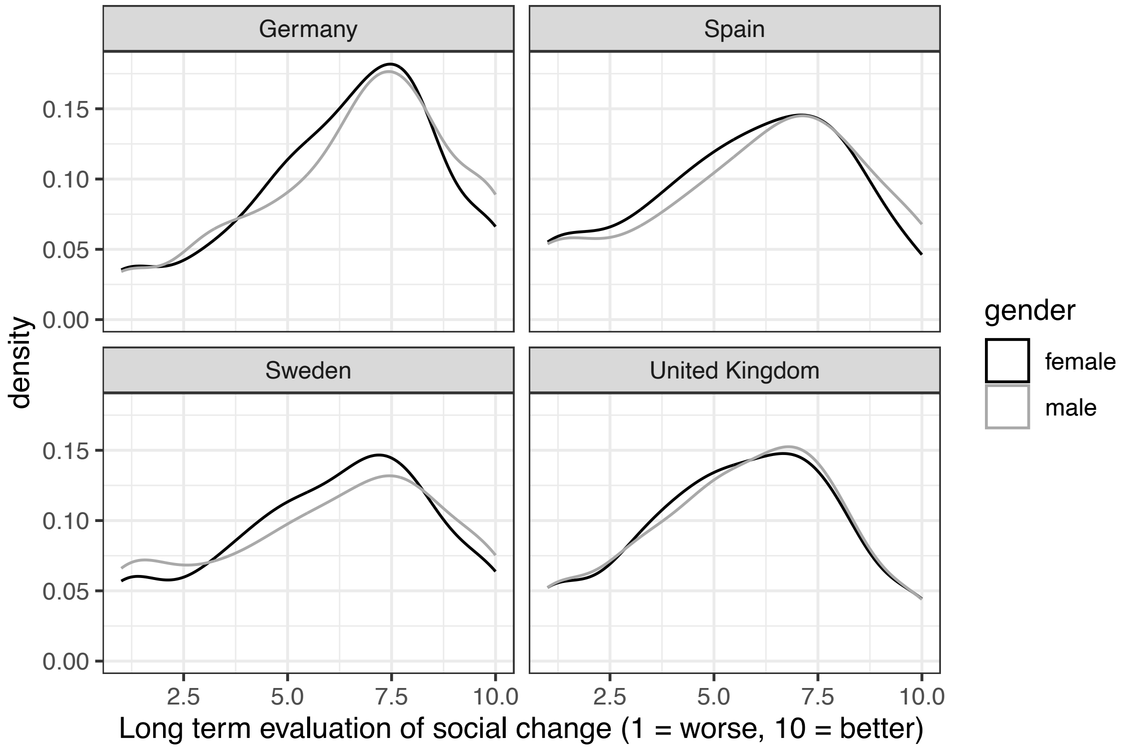

Figure B.3: Evaluations of social change

C. Predictors of mobility perceptions

All subsequent analyses control for age and include country fixed effects.

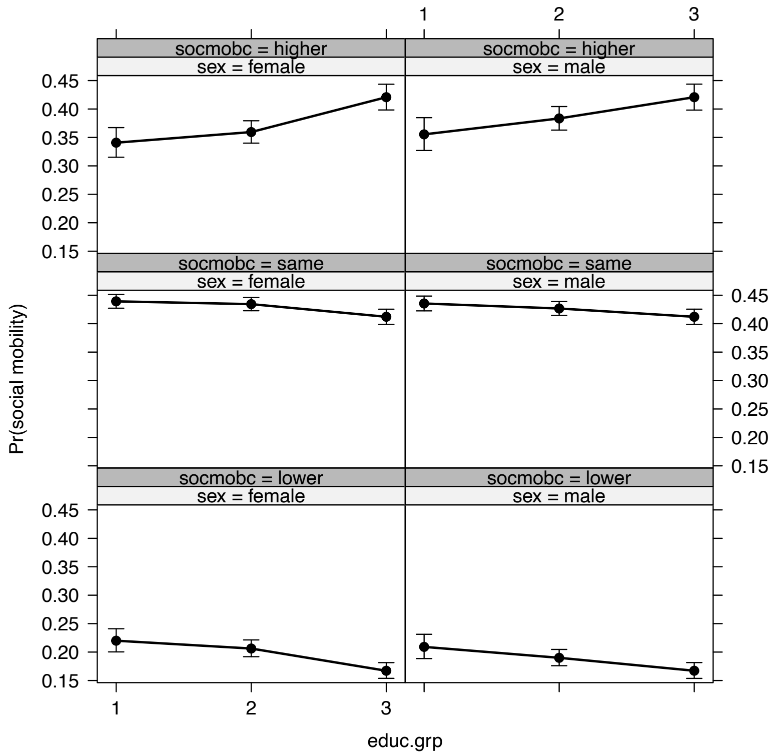

Figure C.1: Education as a predictor of social mobility compared to parents

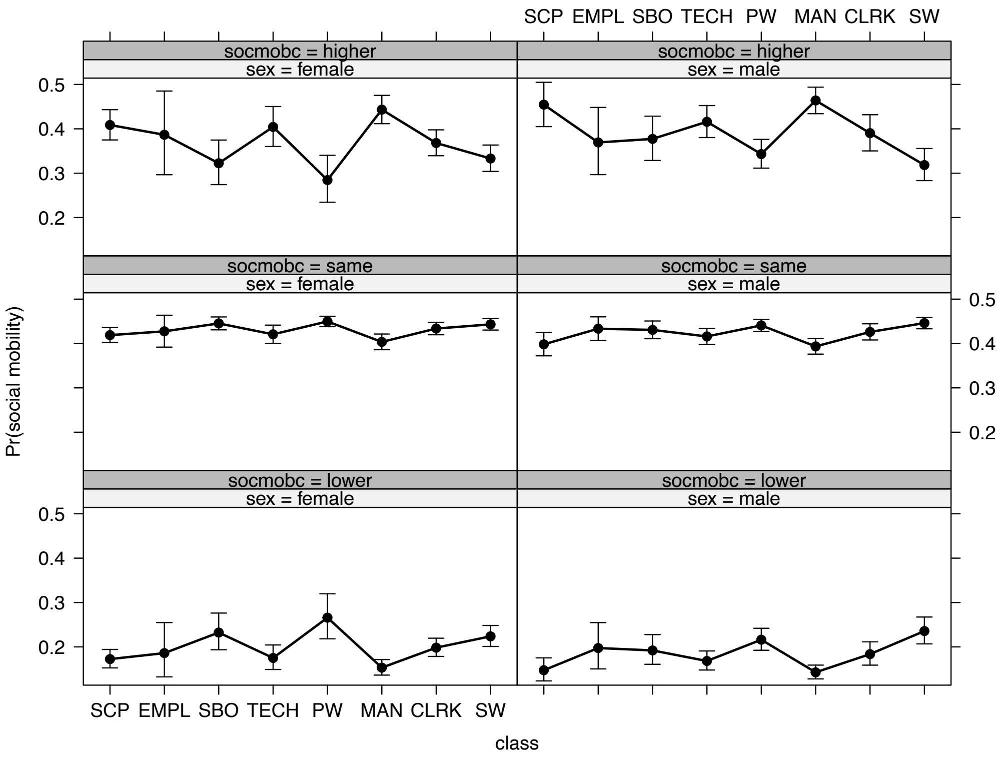

Figure C.2: Oesch class as a predictor of social mobility compared to parents

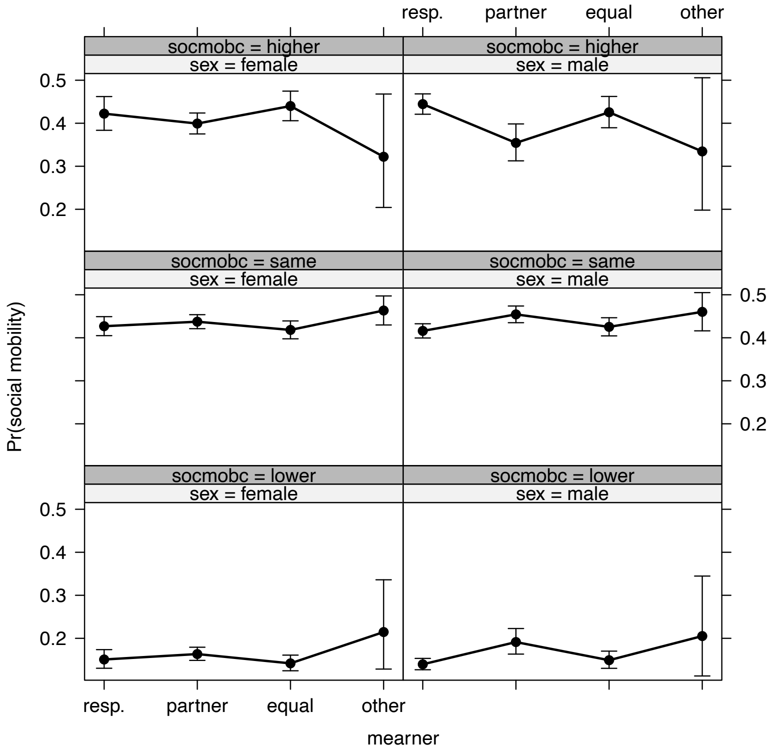

Figure C.3: Main household earner as a predictor of social mobility compared to parents

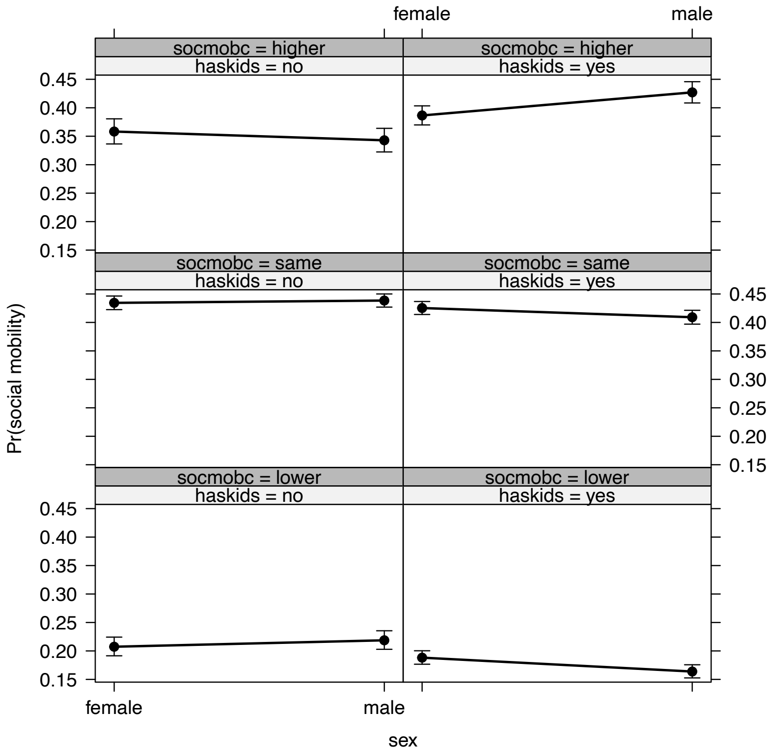

Figure C.4: Children as a predictor of social mobility compared to parents

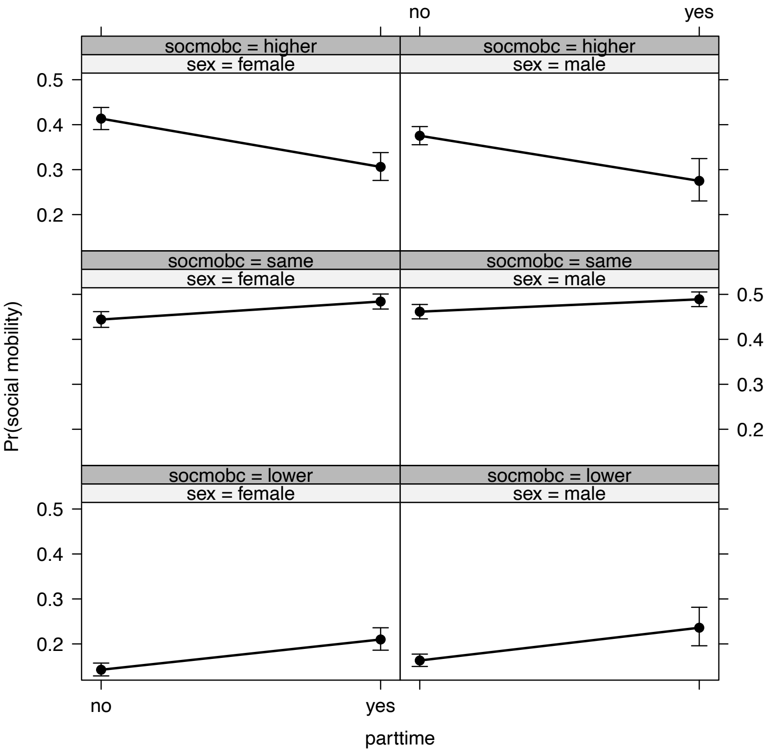

Figure C.5: Part-time employment as a predictor of social mobility compared to parents

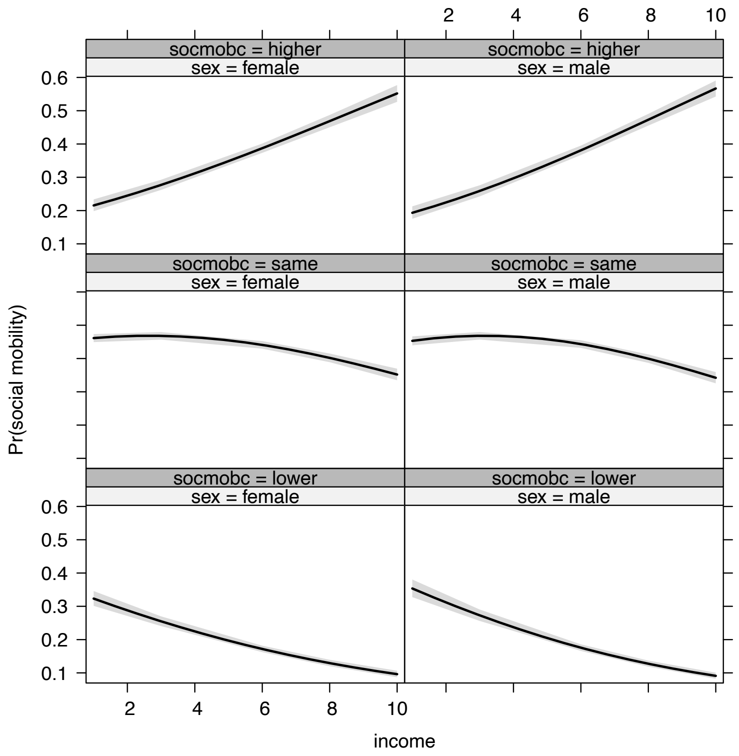

Figure C.6: Income (deciles) as a predictor of social mobility compared to parents

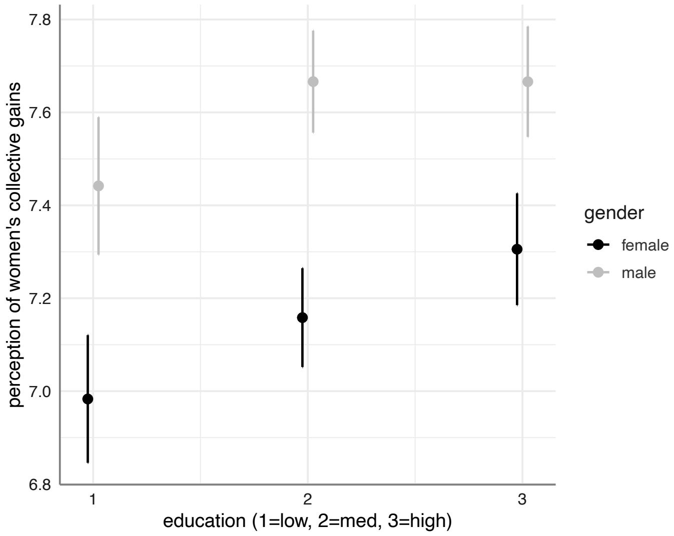

Figure C.7: Education as a predictor of women's perceived gains

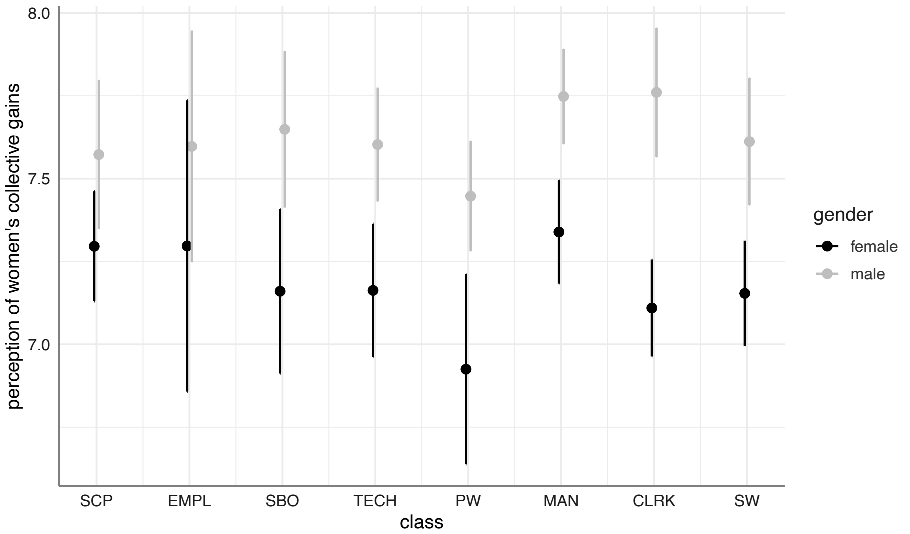

Figure C.8: Class (Oesch) as a predictor of women's perceived gains

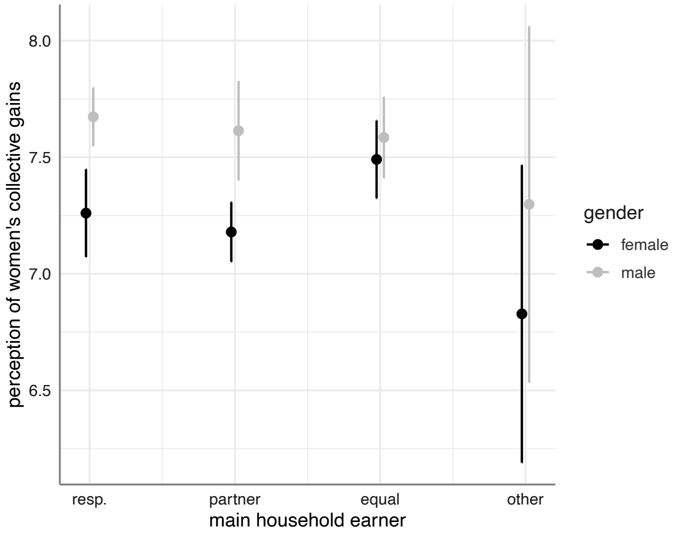

Figure C.9: Main household earner as a predictor of women's perceived gains

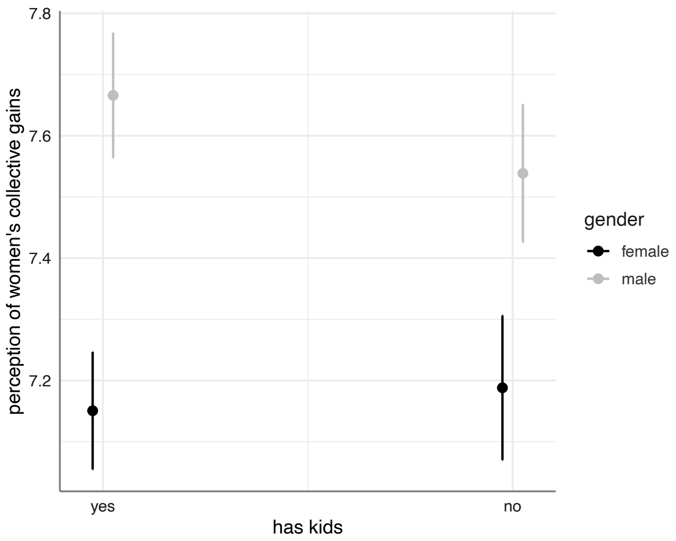

Figure C.10: Children as a predictor of women's perceived gains

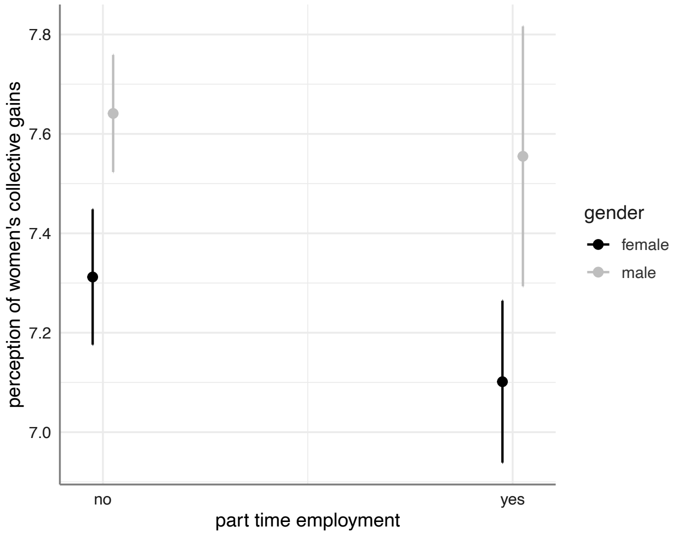

Figure C.11: Part-time employment as a predictor of women's perceived gains

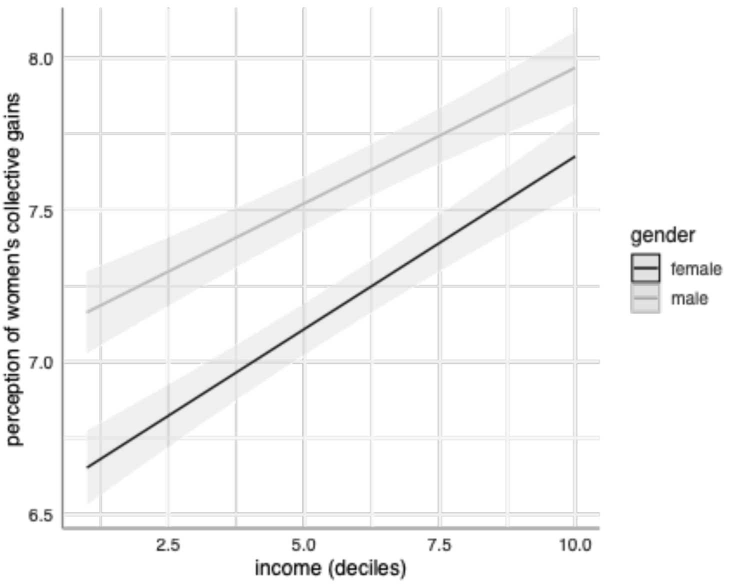

Figure C.12: Income (deciles) as a predictor of women's perceived gains

D. Regression Tables and Additional Figures (Main Results)

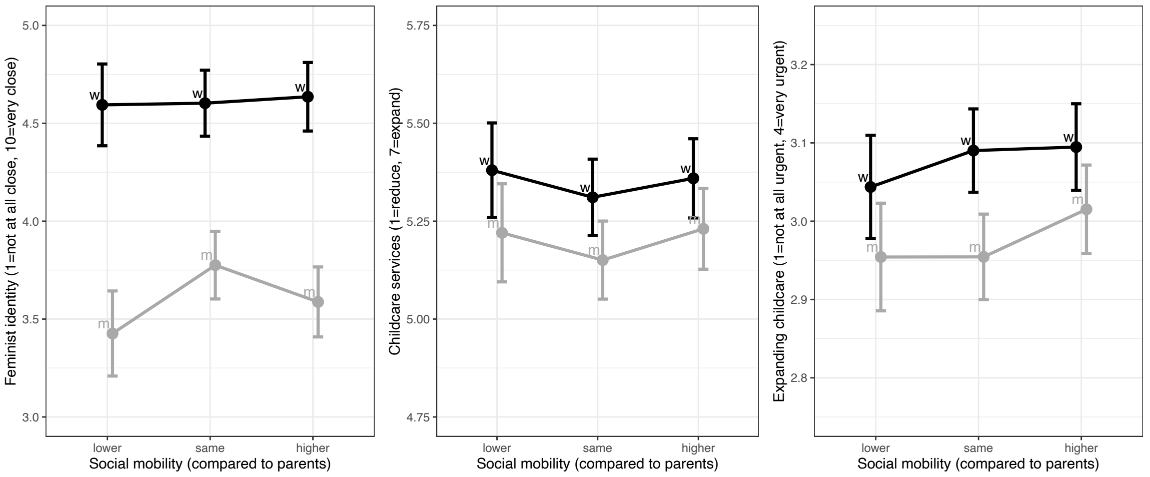

Figure D.1: Perceptions of upward individual mobility not associated with progressivism on gender equality among women

Note: OLS regressions controlling for age and education, with country FE.

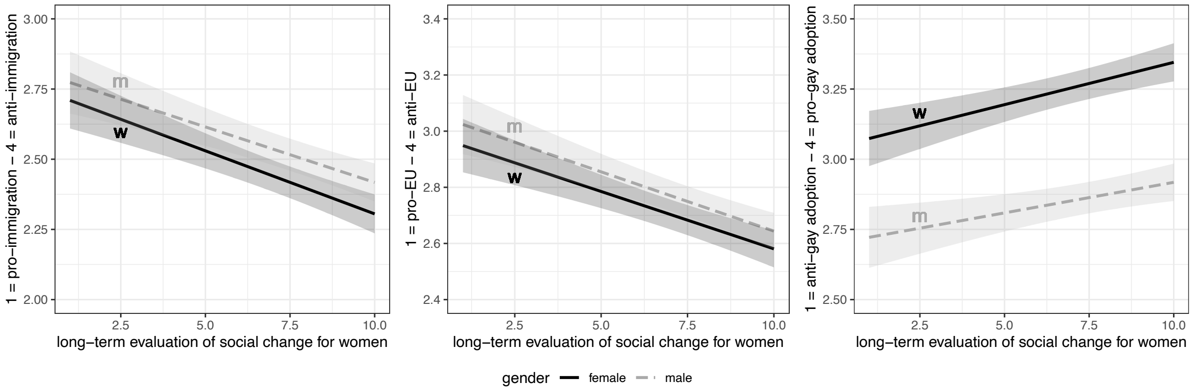

Figure D.2: Views of women's collective mobility not *especially* positively associated with general progressivism among women (no more so than among men)

Note: OLS regressions controlling for age and education, with country FE.

Table D1: Regressions: Opportunity Perceptions (Figures 4 and 5)

	Fig 4, l	Fig 4, r	Fig 5, l	Fig 5, r	rob a	rob b
Intercept	4.22***	4.08***	3.16***	3.34***	2.55***	2.39***
	(0.17)	(0.17)	(0.22)	(0.19)	(0.22)	(0.22)
male	0.37*	0.38*	1.13***	0.43*	0.96***	0.96***
	(0.15)	(0.16)	(0.25)	(0.17)	(0.25)	(0.25)
medium education	0.66***	0.65***	0.69***	0.62***	0.63***	0.61***
	(0.09)	(0.09)	(0.10)	(0.09)	(0.09)	(0.09)
high education	1.07***	1.03***	1.17***	1.04***	1.03***	0.99***
	(0.10)	(0.10)	(0.10)	(0.10)	(0.10)	(0.10)
age	−0.00*	−0.01*	−0.00	0.00	−0.00	−0.01*
	(0.00)	(0.00)	(0.00)	(0.00)	(0.00)	(0.00)
mobility=parents	1.57***				1.43***	
	(0.12)				(0.09)	
mobility>parents	2.26***				2.00***	
	(0.13)				(0.10)	
male*mobility=parents	−0.08					
	(0.18)					
male*mobility>parents	0.01					
	(0.19)					
mobility=mother		1.64***				1.54***
		(0.13)				(0.10)
mobility>mother		2.34***				2.10***
		(0.13)				(0.10)
male*mobility=mother		−0.06				
		(0.19)				
male*mobility>mother		−0.00				
		(0.19)				
eval change women			0.34***		0.26***	0.26***
			(0.02)		(0.02)	(0.02)
male*eval change women			−0.12***		−0.10**	−0.10**
			(0.03)		(0.03)	(0.03)
eval social change				0.34***		
				(0.02)		
male*eval social change				−0.01		
				(0.03)		
R^2	0.17	0.18	0.14	0.18	0.20	0.20
Adj. R^2	0.17	0.18	0.14	0.18	0.19	0.20
Num. obs.	6570	6570	6570	6558	6521	6523
Country fixed effects	yes	yes	yes	yes	yes	yes

$^{***}p < 0.001$; $^{**}p < 0.01$; $^{*}p < 0.05$

Note: The last two models show that the interaction effect between gender and evaluations for social change for women holds when we control for individual mobility experiences (compared to parents as well as to mothers).

Table D2: Regressions: socio-cultural attitudes and individual trajectory (Figure 6)

	Anti-immigrant	Anti-EU	Pro-gay adoption
Intercept	2.26***	2.56***	3.87***
	(0.05)	(0.05)	(0.05)
medium education	−0.18***	−0.13***	0.03
	(0.03)	(0.03)	(0.03)
high education	−0.39***	−0.34***	0.02
	(0.03)	(0.03)	(0.03)
age	0.00***	0.00***	−0.01***
	(0.00)	(0.00)	(0.00)
male	0.13**	0.09*	−0.45***
	(0.05)	(0.05)	(0.05)
mobility=parents	−0.05	−0.10*	−0.05
	(0.04)	(0.04)	(0.04)
mobility>parents	−0.07	−0.10**	0.01
	(0.04)	(0.04)	(0.04)
male*mobility=parents	0.03	−0.01	0.07
	(0.06)	(0.05)	(0.06)
male*mobility>parents	−0.15*	−0.09	0.08
	(0.06)	(0.06)	(0.06)
R^2	0.04	0.04	0.08
Adj. R^2	0.04	0.04	0.08
Num. obs.	8948	8943	8624
Country fixed effects	yes	yes	yes

$^{***}p < 0.001$; $^{**}p < 0.01$; $^{*}p < 0.05$

Table D3: Regressions: Gender attitudes and collective trajectory (Figure 7)

	Feminist ID	Expand childcare	Prioritize childcare
Intercept	5.20***	5.31***	3.16***
	(0.17)	(0.10)	(0.05)
medium education	0.09	−0.05	−0.05*
	(0.07)	(0.04)	(0.02)
high education	0.48***	−0.14**	−0.06*
	(0.08)	(0.04)	(0.02)
age	−0.02***	−0.00***	−0.00***
	(0.00)	(0.00)	(0.00)
male	−0.42*	0.13	0.08
	(0.20)	(0.12)	(0.06)
eval change women	0.05**	0.03**	0.01*
	(0.02)	(0.01)	(0.01)
male*eval change women	−0.08**	−0.04**	−0.03**
	(0.03)	(0.02)	(0.01)
R^2	0.07	0.03	0.03
Adj. R^2	0.07	0.03	0.03
Num. obs.	8897	8769	8915
Country fixed effects	yes	yes	yes

$^{***}p < 0.001$; $^{**}p < 0.01$; $^{*}p < 0.05$

Table D4: Regressions: Gender attitudes and individual trajectory (Figure D.1)

	Feminist ID	Expand childcare	Prioritize childcare
Intercept	5.49^{***}	5.56^{***}	3.23^{***}
	(0.14)	(0.08)	(0.04)
medium education	0.09	−0.06	-0.05^{*}
	(0.07)	(0.04)	(0.02)
high education	0.49^{***}	-0.14^{**}	-0.07^{**}
	(0.08)	(0.04)	(0.02)
age	-0.02^{***}	-0.00^{***}	-0.00^{***}
	(0.00)	(0.00)	(0.00)
male	-1.17^{***}	-0.16^{*}	-0.09^{*}
	(0.12)	(0.07)	(0.04)
mobility=parents	0.01	−0.07	0.05
	(0.10)	(0.06)	(0.03)
mobility>parents	0.04	−0.02	0.05
	(0.11)	(0.06)	(0.03)
male*mobility=parents	0.34^{*}	−0.00	−0.05
	(0.15)	(0.09)	(0.05)
male*mobility>parents	0.12	0.03	0.01
	(0.15)	(0.09)	(0.05)
R^2	0.07	0.03	0.03
Adj. R^2	0.07	0.03	0.03
Num. obs.	8906	8779	8925
Country fixed effects	yes	yes	yes

$^{***}p < 0.001$; $^{**}p < 0.01$; $^{*}p < 0.05$

Table D5: Regressions: socio-cultural attitudes and collective trajectory (Figure D.2)

	Anti-immigrant	Anti-EU	Pro-gay adoption
Intercept	2.55^{***}	2.79^{***}	3.63^{***}
	(0.07)	(0.06)	(0.06)
medium education	-0.18^{***}	-0.13^{***}	0.03
	(0.03)	(0.03)	(0.03)
high education	-0.39^{***}	-0.33^{***}	0.02
	(0.03)	(0.03)	(0.03)
age	0.00^{***}	0.00^{***}	-0.01^{***}
	(0.00)	(0.00)	(0.00)
male	0.06	0.08	-0.34^{***}
	(0.08)	(0.07)	(0.08)
eval change women	-0.04^{***}	-0.04^{***}	0.03^{***}
	(0.01)	(0.01)	(0.01)
male*eval change women	0.01	−0.00	−0.01
	(0.01)	(0.01)	(0.01)
R^2	0.04	0.05	0.09
Adj. R^2	0.04	0.05	0.09
Num. obs.	8937	8932	8614
Country fixed effects	yes	yes	yes

$^{***}p < 0.001$; $^{**}p < 0.01$; $^{*}p < 0.05$

Table D6: Regressions: Gender attitudes and collective trajectory, controlling for individual trajectory (compared to parents)

	Feminist ID	Expand childcare	Prioritize childcare
Intercept	5.10^{***}	5.37^{***}	3.16^{***}
	(0.18)	(0.10)	(0.06)
medium education	0.09	-0.06	-0.05^{*}
	(0.07)	(0.04)	(0.02)
high education	0.48^{***}	-0.14^{**}	-0.06^{**}
	(0.08)	(0.04)	(0.02)
age	-0.02^{***}	-0.00^{***}	-0.00^{***}
	(0.00)	(0.00)	(0.00)
male	-0.47^{*}	0.14	0.06
	(0.20)	(0.12)	(0.06)
mobility>parents	0.15	-0.08	0.02
	(0.08)	(0.04)	(0.02)
mobility=parents	0.07	-0.01	0.06^{*}
	(0.08)	(0.05)	(0.02)
eval change women	0.05^{*}	0.03^{*}	0.01
	(0.02)	(0.01)	(0.01)
male*eval change women	-0.07^{**}	-0.04^{**}	-0.02^{**}
	(0.03)	(0.02)	(0.01)
R^2	0.08	0.03	0.03
Adj. R^2	0.07	0.03	0.03
Num. obs.	8828	8701	8845
Country fixed effects	yes	yes	yes

$^{***}p < 0.001$; $^{**}p < 0.01$; $^{*}p < 0.05$

Table D7: Collective evaluations X individual mobility on outcomes among women

	Opportunities	Feminist ID	Expand childcare	Prioritize childcare
Intercept	2.86^{***}	5.31^{***}	5.46^{***}	3.30^{***}
	(0.34)	(0.28)	(0.16)	(0.09)
medium education	0.70^{***}	0.17	-0.08	-0.07^{*}
	(0.13)	(0.10)	(0.06)	(0.03)
high education	1.11^{***}	0.67^{***}	-0.18^{**}	-0.06^{*}
	(0.13)	(0.10)	(0.06)	(0.03)
age	-0.01^{*}	-0.02^{***}	-0.00^{***}	-0.00^{***}
	(0.00)	(0.00)	(0.00)	(0.00)
eval change women	0.21^{***}	0.07^{*}	0.02	-0.01
	(0.04)	(0.04)	(0.02)	(0.01)
mobility=parents	0.64	-0.16	-0.17	-0.13
	(0.39)	(0.32)	(0.19)	(0.10)
mobility>parents	1.96^{***}	0.69	-0.10	-0.19
	(0.46)	(0.36)	(0.21)	(0.11)
eval change women*mobility=parents	0.11^{*}	0.01	0.01	0.02
	(0.06)	(0.05)	(0.03)	(0.01)
eval change women*mobility>parents	-0.00	-0.10^{*}	0.01	0.03^{*}
	(0.06)	(0.05)	(0.03)	(0.02)
R^2	0.22	0.06	0.03	0.03
Adj. R^2	0.21	0.05	0.02	0.03
Num. obs.	3363	4574	4487	4588
Country fixed effects	yes	yes	yes	yes

$^{***}p < 0.001$; $^{**}p < 0.01$; $^{*}p < 0.05$

E. Robustness checks: Meritocratic beliefs

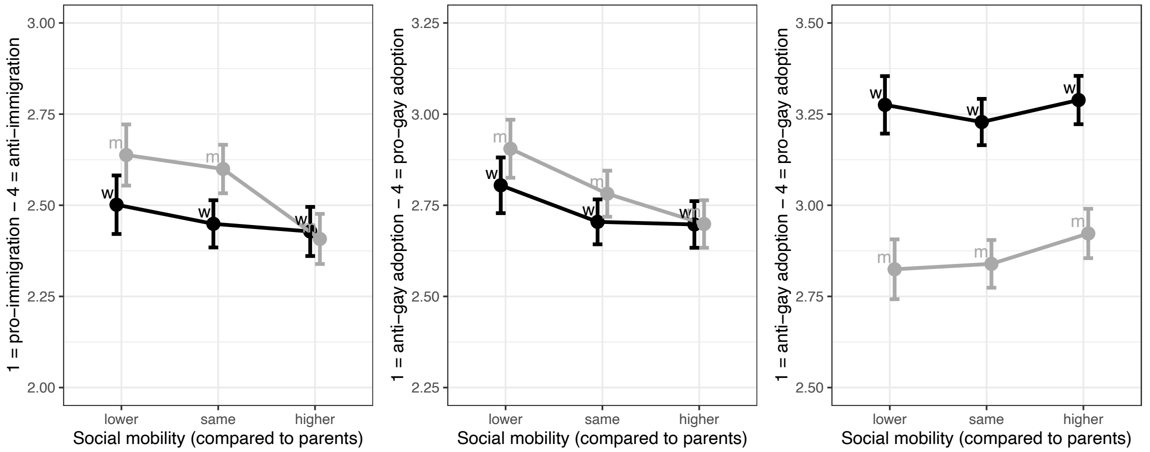

Figure E.1: Perceptions of upward individual mobility not associated with general progressivism among women when controlling for meritocratic beliefs

OLS regressions controlling for age, education, and meritocracy beliefs, with country FE.

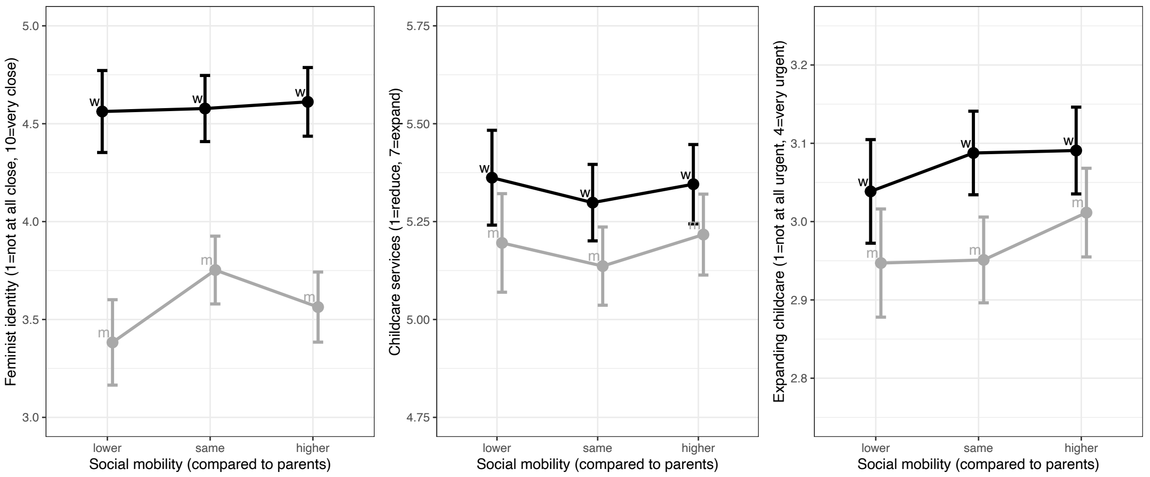

Figure E.2: Perceptions of upward individual mobility not associated with progressivism on gender equality among women when controlling for meritocratic beliefs

Note: OLS regressions controlling for age, education, and meritocracy beliefs, with country FE.
